# High Responsivity Thermopile Sensors Featuring a Mosaic Structure

**DOI:** 10.3390/mi13060934

**Published:** 2022-06-11

**Authors:** Elisabetta Moisello, Maria Eloisa Castagna, Antonella La Malfa, Giuseppe Bruno, Piero Malcovati, Edoardo Bonizzoni

**Affiliations:** 1 Department of Electrical, Computer and Biomedical Engineering, University of Pavia, Via Ferrata 5, 2100 Pavia, Italy; piero.malcovati@unipv.it (P.M.); edoardo.bonizzoni@unipv.it (E.B.); 2 STMicroelectronics, 95121 Catania, Italy; mariaeloisa.castagna@st.com (M.E.C.); antonella.lamalfa@st.com (A.L.M.); giuseppe.bruno@st.com (G.B.)

**Keywords:** thermopile, TMOS, thermal sensor, proximity, motion, temperature

## Abstract

This paper presents a detailed analysis of a micromachined thermopile detector featuring high responsivity and a versatile mosaic structure, based on 128 60 µm × 60 µm pixels connected in series and/or in parallel. The mosaic structure is based on the one employed for the thermal sensor known as TMOS, which consists of a CMOS-SOI transistor embedded in a suspended and thermally isolated absorbing membrane, released through microelectro mechanical system (MEMS) post-processing. Two versions of the thermopile detector, featuring different series/parallel connections, are presented and were experimentally characterized. The most performant of the two achieved 2.7 × 10${}^{4}$ V/W responsivity. The thermopile sensors’ performances are compared to that of the TMOS sensor, adopting different configurations, and their application as proximity detectors was verified through measurements.

## 1. Introduction

In the last few years, the research interest in miniaturized low-power thermal detectors has greatly increased, due to the spread of Internet of Things (IoT) and portable devices, and due to the need for contactless temperature checks and appliances operation (i.e., sanitizing gel dispensers) during the COVID-19 pandemic.

There exist different types of thermal detectors: bolometers, pyroelectric detectors (PIR), thermopiles and the recently developed TMOS sensor. All thermal detectors rely on the Stefan–Boltzmann law, which states that every object emits thermal radiation depending on its temperature, thereby enabling contactless temperature measurements. Among thermal detectors, thermopiles [1,2,3,4,5] and TMOS [6,7,8,9] have emerged as the preferred ones, as they feature the best combination of performance, power consumption and cost [10]. Indeed, bolometers are not compatible with standard CMOS processes, thereby entailing higher costs, and PIR detectors are inherently AC devices and require additional optical or mechanical chopping to perform contactless tempearture measurements, thereby significantly adding to the power consumption and the overall sensor size. Instead, thermopiles are self-biased, and therefore feature the best power consumption while offering good performance. TMOS offers by far the best performance (i.e., the highest responsivity and sensitivity values), while featuring very limited power consumption thanks to the transistor’s operation at a subthreshold level. Both thermopiles and TMOS are fully compatible with standard CMOS-SOI processes, enabling large-volume fabrication at low-cost.

Recently a novel type of thermopile detector, which exploits a mosaic structure analogous to the one of the TMOS sensor in order to obtain high responsivity, has been presented [11].

This paper proposes a supplementary in-depth analysis of the thermopile detectors proposed in [11], focusing in particular on a comparison with the TMOS sensor.

The paper is organized as follows. Section 2 illustrates the characteristics and biasing configurations of the employed TMOS sensors, and Section 3 provides a detailed characterization of the proposed thermopile detectors performance. Section 4 features the comparison between the TMOS and the thermopile sensor, considering in particular the case of proximity detection. Section 5 concludes the paper.

## 2. TMOS

**Figure 1:**
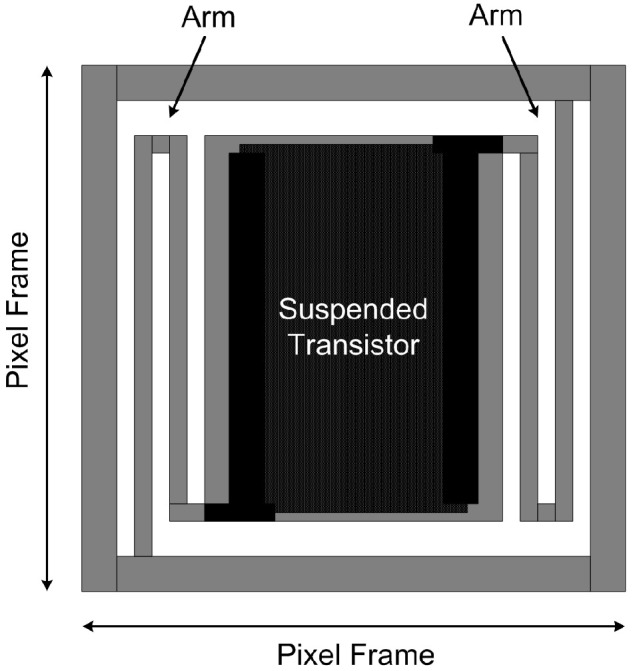
Schematic representation of the TMOS sensor pixel.

**Figure 2:**
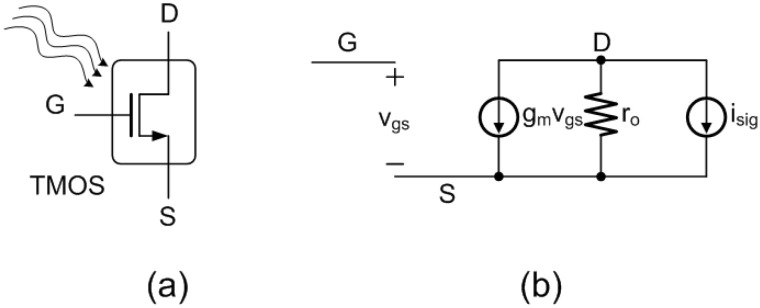
Equivalent NMOS transistor (**a**) symbol and (**b**) small-signal circuit model.

The TMOS sensor is based on a multi-pixel mosaic structure, with each pixel featuring a 130 nm CMOS-SOI transistor embedded in a suspended and thermally isolated absorbing membrane, obtained through MEMS post processing. As the membrane absorbs thermal radiation from the target object and the surroundings, the transistor temperature is varied, thereby modifying its I–V characteristics and generating a signal. A schematic representation of the TMOS pixel is illustrated in Figure 1. Each pixel features a CMOS-SOI NMOS transistor with 77.4 µm width and 15.8 µm length. The TMOS mosaic structure features 128 60 µm × 60 µm pixels, which form two 8 × 8 matrices: each matrix, therefore, consists of 64 pixels connected in parallel, which act as an equivalent 130-nm CMOS-SOI NMOS transistor, as modeled schematically in Figure 2 [8,9]. The equivalent CMOS-SOI NMOS transistors feature a length equal to the one of the single pixel and a total width equal to that of a single pixel times the number of pixels connected in parallel, i.e., 64. One matrix constitutes the active device, exposed to the target object thermal radiation, and the other matrix constitutes the blind device, shielded by an aluminum mirror, and therefore only able to see itself and act as a reference. A pair of devices, one active and one blind, is employed in order to cancel out common-mode contributions, both thermal and electrical: indeed, the differential voltage between the drain terminals of the equivalent transistor of the active and blind devices constitutes the TMOS sensor output signal. The TMOS fabrication process and packaging steps can be found at [12].

**Figure 3:**
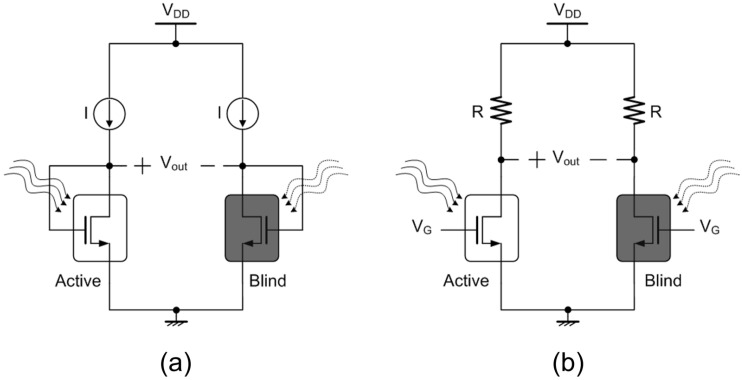
Schematic representations of the considered (**a**) 2T and (**b**) 3T TMOS configurations [8].

The TMOS performance strongly depends on the transistor operating point and configuration. The devices are biased in subthreshold region, which ensures the highest sensitivity, as the operation is based on diffusion, which is more sensitive to temperature. Two main different device configurations are considered: two-terminal diode-like (2–T) and three-terminal (3–T) configuration [8]. The considered 2–T and 3–T configurations are illustrated schematically in Figure 3a and Figure 3b, respectively.

For both configurations, supposing subthreshold operation and the drain-to-source voltage $V_{DS}$ larger than a few $\frac{kT}{q}$ (i.e., the thermal voltage), the drain-to-source current, $I_{DS}$, is expressed as

(1) $$I_{DS}=I_{D0}\;e^{\frac{q(V_{GS}-V_{T})}{nkT}}$$

which yields a current sensitivity with respect to the TMOS temperature variation, $S_{I,TMOS}$, equal to:

(2) $$S_{I,TMOS}=\frac{dI_{DS}}{dT}=-I_{DS}\frac{q}{nkT}\left(\frac{dV_{T}}{dT}+\frac{V_{GS}-V_{T}}{T}\right)$$

The current sensitivity with respect to the TMOS temperature variation, $S_{I,TMOS}$, can be converted into the voltage sensitivity with respect to the TMOS temperature variation, $S_{V,TMOS}$, according to

(3) $$S_{V,TMOS}=-Z_{out}\;S_{I,TMOS}$$

where $Z_{out}$ is the circuit output impedance [9]. In the case of the 2–T configuration, $Z_{out}$ is equal to $1/g_{m,TMOS}$, and for the 3–T configuration $Z_{out}$ is equal to *R*, provided that $R>>r_{o}$.

The TMOS sensitivity to the target temperature variation can be expressed as

(4) $$S_{V,target}=\frac{\Delta T_{TMOS}}{\Delta T_{target}}\;S_{V,TMOS}$$

where Δ$T_{TMOS}$ is the temperature variation induced on the sensor and $\Delta T_{target}$ the temperature difference between the target and the ambient. The TMOS sensor output voltage, therefore, can be derived as

(5) $$V_{out}=S_{V,TMOS}\;\Delta T_{TMOS}=S_{V,target}\;\Delta T_{target}$$

Δ$T_{TMOS}$, can be calculated as

(6) $$\Delta T_{TMOS}=\frac{P_{in}}{G_{th}}$$

where $P_{in}$ is the incident radiant power falling on the detector and $G_{th}$ the TMOS thermal conductance, equal to 8.5 ×$10^{-8}$ W/K.

$P_{in}$ is calculated as

(7) $$P_{in}\left(T_{s},T_{d}\right)=\frac{\sigma \epsilon_{s}\epsilon_{d}A_{s}F_{sd}}{\pi N}\left(T_{s}^{4}-T_{d}^{4}\right)$$

where σ is the Stefan–Boltzmann constant, $\epsilon_{s}$ the source object emissivity, $\epsilon_{d}$ the TMOS emissivity, $A_{s}$ the source object area, $T_{s}$ the target object temperature, $T_{d}$ the detector temperature, $F_{sd}$ a transfer factor which takes into account the detector-source system geometry and *N* the number of pixels of the TMOS matrix, i.e., 64 [9].

The performance of the considered TMOS circuit configurations was evaluated by means of Cadence Virtuoso simulations, by varying the local temperature of the active equivalent NMOS device. The temperature variation induced on the sensor, Δ$T_{TMOS}$, was calculated relying on (6) and (7).

Simulations were performed with various biasing currents of the transistors by directly modifying current generator *I* in the 2–T configuration and by tuning $V_{G}$ in the 3–T configuration. Furthermore, in the 3–T circuit, resistance value *R* was adjusted in order to maintain the output common-mode voltage equal to $V_{DD}/2$, i.e., 600 mV.

**Figure 4:**
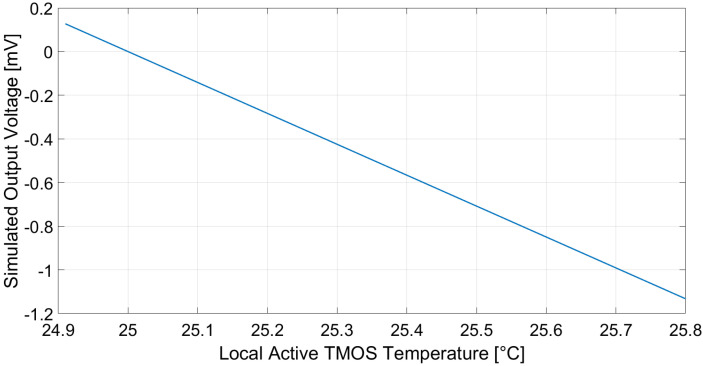
Simulated 2–T configuration TMOS sensor output voltage. Cadence Virtuoso was employed as simulation software.

**Figure 5:**
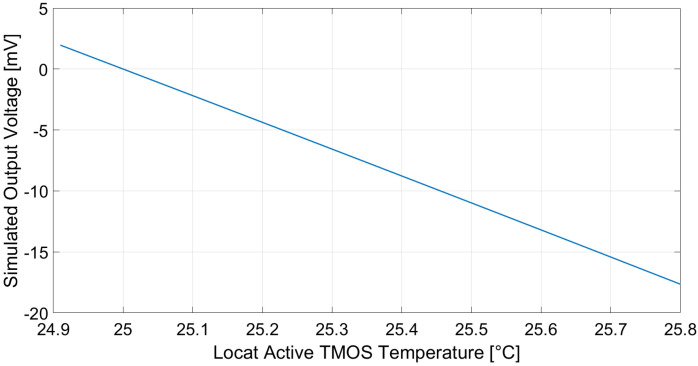
Simulated 3–T configuration TMOS sensor output voltage. Cadence Virtuoso was employed as simulation software.

The simulated output voltages considering the 2–T and 3–T configurations, while supposing a 1-µA biasing current, an ambient temperature equal to 25 °C and a 10 cm × 10 cm black body at 10 cm distance with a temperature varying from 20 to 60 °C as the target object, are reported in Figure 4 and Figure 5, respectively. Employing (4), the 2–T and 3–T voltage sensitivities to the target temperature are –28 and –456 µV/°C, respectively. Furthermore, the sensor responsivity, defined as $|V_{out}|$/$P_{in}$, is equal to 1.14 × 10${}^{4}$ and 1.83 × 10${}^{5}$ V/W for the 2–T and 3–T circuits, respectively.

Analogous simulations and calculations were performed for different biasing current values: the derived sensitivity and responsivity values are illustrated, respectively, in Table 1 and Table 2.

## 3. Thermopile Sensor

**Figure 6:**
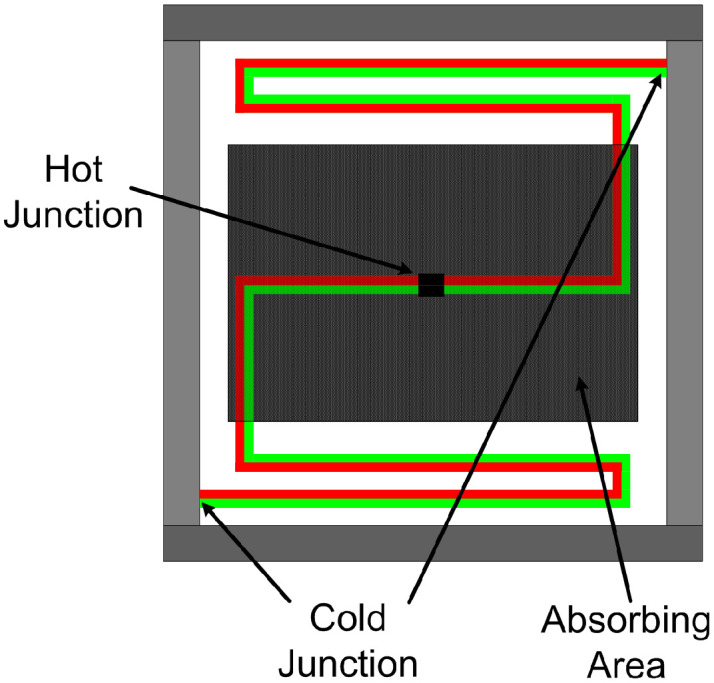
Schematic representation of the proposed thermopile pixel.

The same 128 60 µm × 60 µm pixels mosaic structure, employed for TMOS, was adopted for the proposed thermopile detector [11]. The basic pixel schematic view is illustrated in Figure 6: it consists of two thermocouple elements placed in parallel, acting as an equivalent thermopile with 7.4 kΩ pixel resistance, equal to the parallel of the two branches resistances. One thermocouple element is realized with n-doped and p-doped polysilicon, and the other is fabricated with n-plus and p-plus wells as conductor materials. The thermocouple joined end (i.e., the hot junction) is embedded in a dielectric membrane, suspended and thermally isolated analogously to the one of the TMOS pixel, which absorbs thermal radiation from the given target object and surroundings.

**Figure 7:**
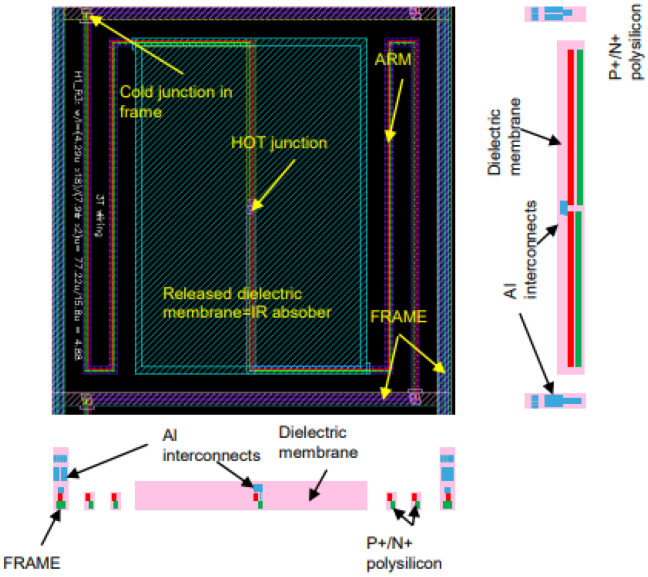
Layout and cross-sectional views of the proposed thermopile pixel.

The proposed thermopile is fabricated by employing the same 130-nm CMOS-SOI technology used for the TMOS sensor. Aluminum layers provide built-in masks for the MEMS micromachining: indeed they act as hard masks during the reactive ion etching (RIE) process, both isotropic and anisotropic, employed for front-side dielectrics removal. Wafer to wafer bonding is used for the top cap wafer, and backside deep RIE of the silicon allows the manufacturing of the suspended pixel and arms. The finished layout and cross-sectional views of the proposed sensors are reported in Figure 7.

The mosaic structure allows excellent versatility: indeed, two thermopiles, referred to as E1 and E2, featuring different series/parallel pixels connections, were fabricated. E1 features 16 elements placed in series, each made up of a series of two sub-elements, where a sub-element consists of 4 pixels connected in parallel: this structure results in 8 equivalent pixels. E2, instead, consists of 128 equivalent pixels, as the 128 pixels are all connected in parallel.

**Figure 8:**
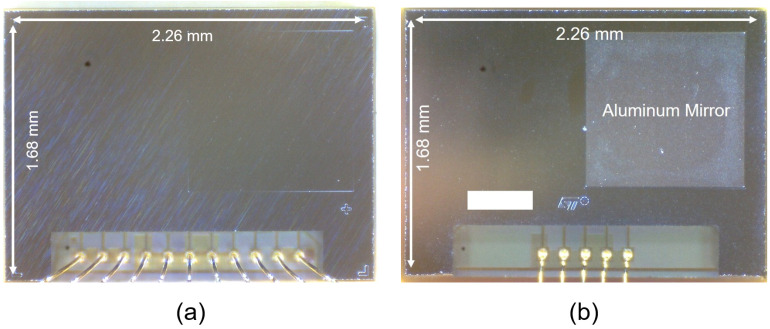
Micrograph of (**a**) the proposed thermopile and (**b**) TMOS packages.

As for TMOS, the proposed thermopile detectors are packaged under vacuum in order to improve the sensor efficiency by eliminating thermal losses due to conduction. Micrographs of the thermopile and TMOS cap packages are reported, respectively, in Figure 8a and Figure 8b. The difference between the two packages is given by the presence of the aluminum mirror which covers the matrix implementing the blind device for TMOS, whereas no mirror is present in the thermopile detector, as all pixels are exposed to thermal radiation.

**Figure 9:**
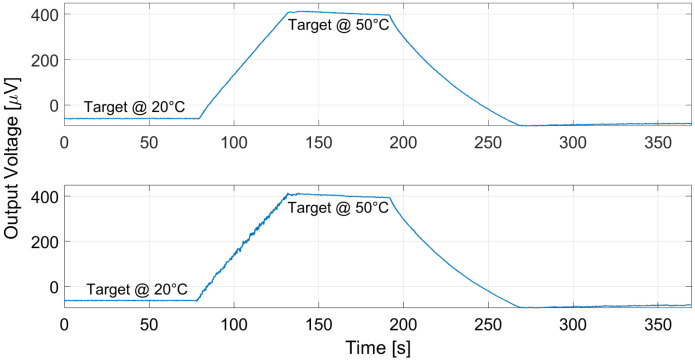
Measured thermopile E1 output with the black body at 10 cm distance and ambient temperature equal to 25 °C. The black body temperature was varied as a ramp from 20 to 50 °C.

**Figure 10:**
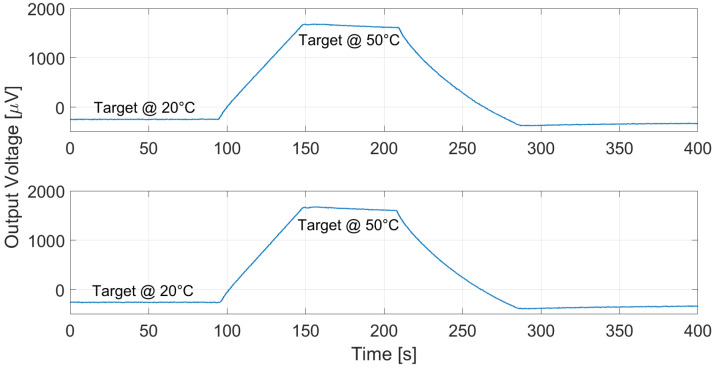
Measured thermopile E2 output with the black body at 10 cm distance and ambient temperature equal to 25 °C. The black body temperature was varied as a ramp from 20 to 50 °C.

The proposed thermopiles, E1 and E2, were characterized considering a 10 cm × 10 cm black body source [13] placed at 10 cm from the sensor. The measurements were performed in a climatic chamber at controlled ambient temperature, equal to 25 °C, while varying the black body temperature in ramp fashion from 20 °C to 50 °C. The measurement results for thermopile E1 and E2 are reported, respectively, in Figure 9 and Figure 10. Multiple measurements were performed and repeatability was verified.

**Figure 11:**
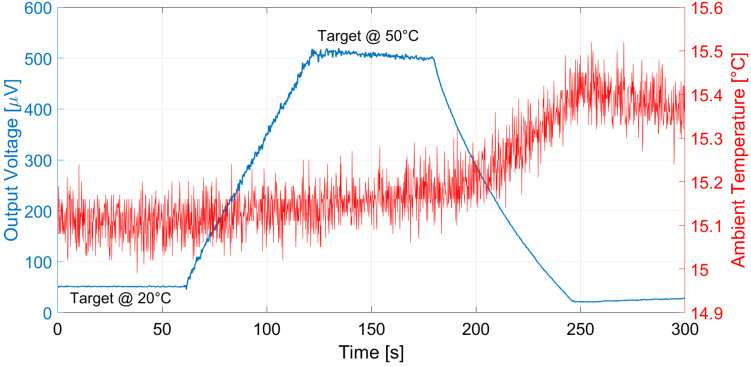
Measured thermopile E1 output with the black body at 10 cm distance and ambient temperature equal to 15 °C. The black body temperature was varied as a ramp from 20 to 50 °C.

**Figure 12:**
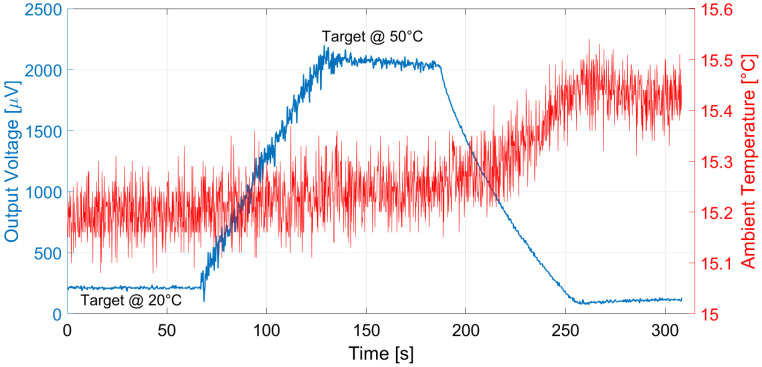
Measured thermopile E2 output with the black body at 10 cm distance and ambient temperature equal to 15 °C. The black body temperature was varied as a ramp from 20 to 50 °C.

**Figure 13:**
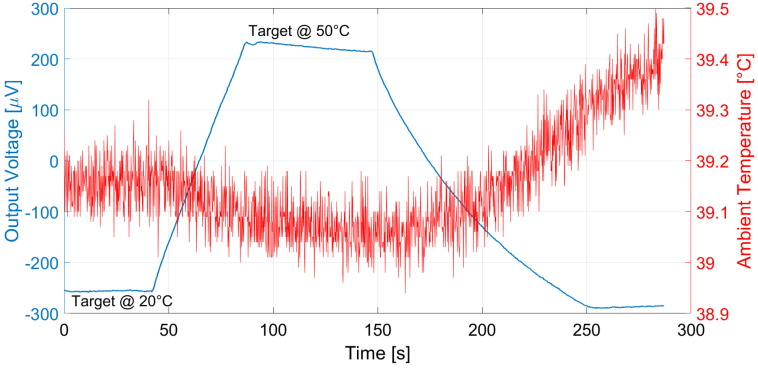
Measured thermopile E1 output with the black body at 10 cm distance and ambient temperature equal to 40 °C. The black body temperature was varied as a ramp from 20 to 50 °C.

**Figure 14:**
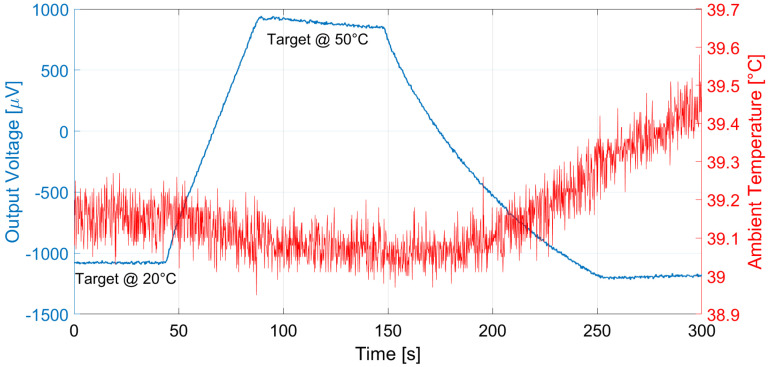
Measured thermopile E2 output with the black body at 10 cm distance and ambient temperature equal to 40 °C. The black body temperature was varied as a ramp from 20 to 50 °C.

Analogous measurements were performed at different ambient temperatures: the results considering a 15 °C ambient temperature for E1 and E2 are reported in Figure 11 and Figure 12, and Figure 13 and Figure 14 illustrate the measurement results for E1 and E2 considering the case of an ambient temperature equal to 40 °C.

The thermopile detector sensitivity was measured for each considered case, both for E1 and E2, as

(8) $$Measured\;Sensitivity=\frac{Output_{Target\;@\;50{\;}^{{}^{\circ}}C}-Output_{Target\;@\;20{\;}^{{}^{\circ}}C}}{50{\;}^{{}^{\circ}}C{}^{\circ}20{\;}^{{}^{\circ}}C}$$

The obtained sensitivity values are reported in Table 3, and Table 4 reports the responsivity values, calculated as

(9) $$Responsivity=\frac{Average\;Sensitivity}{1{\;}^{{}^{\circ}}C\cdot P_{in}}$$

The derived sensitivity and responsivity values differ slightly from the ones reported in [11], as a different range of target object temperatures, resulting in a different linearization, and different thermopile samples have been considered. The obtained responsivity values outperform typical thermopile responsivity by more than one order of magnitude [11], thereby verifying the benefit of the adopted structure.

The noise of the proposed thermopiles was measured by acquiring 5000 output voltage samples at 10 Hz and considering the standard deviation, while maintaining the ambient temperature at 25 °C and the black body at 10 cm distance and 20 °C temperature. The measured noise values were 0.788 µV for E1 and 4.297 µV for E2. These values exceed the electronic noise values, determined solely by the thermopile output resistance thermal noise [11]: this is due to the fact that the electronic noise is not the only noise contribution. Indeed there is also thermal environmental noise, due to the ambient temperature variations (±0.038 °C) and those of the black body source (±0.008 °C). Nevertheless, the measured noise ensures good signal-to-noise ratios equal roughly to 20.

Table 5 summarizes the proposed thermopile detectors characteristics. A detailed comparison with other state-of-the-art thermopile sensors can be found at [11].

## 4. Comparison between Thermopile and TMOS Sensors

**Figure 15:**
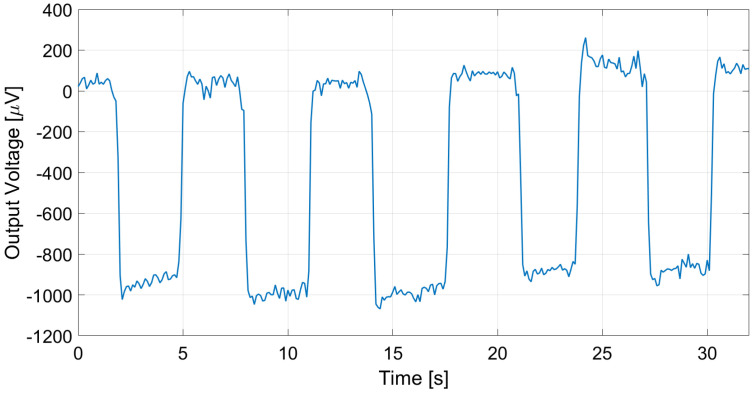
Measured 3–T TMOS output in the case of a hand moved in front of the sensor at 5 cm distance.

**Figure 16:**
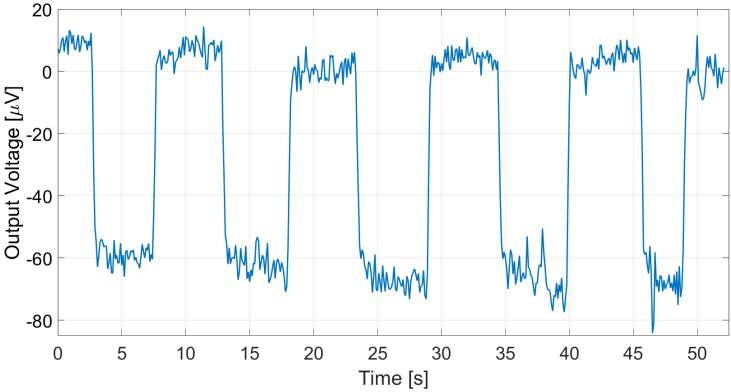
Measured diode–connected TMOS output in the case of a hand moved in front of the sensor at 5 cm distance.

**Figure 17:**
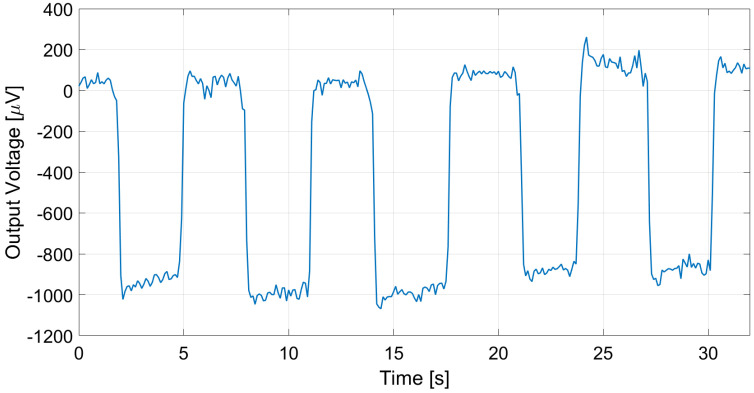
Measured E1 thermopile output in the case of a hand moved in front of the sensor at 5 cm distance.

**Figure 18:**
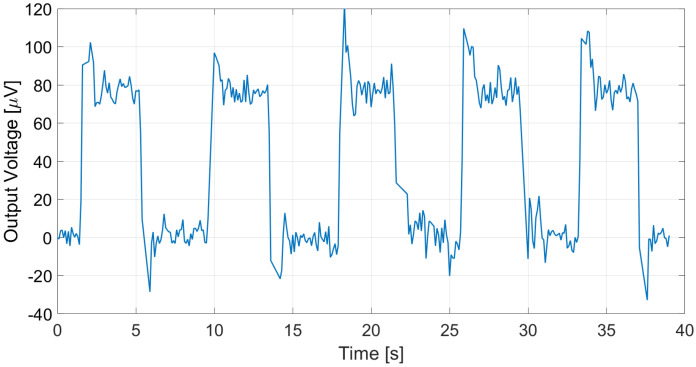
Measured E2 thermopile output in the case of a hand moved in front of the sensor at 5 cm distance.

The proposed thermopile detectors and TMOS, considering both the 2–T and the 3–T configurations, were tested as proximity detectors by moving a hand in front of the sensor at 5 cm distance at 25 °C ambient temperature. The TMOS sensor biasing current was 1 µA for both configurations, and *R* = 600 kΩ for the 3–T case. The measurements results for the 3–T and 2–T TMOS sensors were reported in Figure 15 and Figure 16, and the measurements for E1 and E2 are illustrated in Figure 17 and Figure 18. All the considered detectors represent viable solutions for proximity applications, i.e., for the detection of a hand in order to operate automatically soap and sanitizer gel dispensers, as the signal peaks corresponding to the hand presence are clearly distinguishable.

As expected from the analysis conducted in Section 2 and Section 3, the 3–T TMOS clearly exhibited the best performance: indeed, it exploits the internal gain of the transistor. The proposed thermopile sensor E2, however, outperformed the 2–T TMOS detector (2.70 × 10${}^{4}$ V/W vs. 1.14 × 10${}^{4}$ V/W in terms of responsivity and 70.5 µV/°C vs. 28.3 µV/°C in terms of sensitivity), while featuring the advantage of self-biasing.

## 5. Conclusions

The paper has presented an in-depth analysis of two thermopile sensors employing a mosaic structure analogous to the one of the TMOS sensor, which allows great versatility, as different series/parallel connections can be easily implemented. The thermopile detectors were experimentally characterized: a very high responsivity value was yielded by E2 (2.7 × 10${}^{4}$ V/W), which outperforms typical state-of-the-art thermopiles. Furthermore, the proposed thermopiles have been compared to the TMOS sensor considering both two-terminal and three-terminal configurations. Thermopile E2, although the 3–T TMOS sensor exhibits by far the best performance, represents a valid alternative if biasing circuits should be avoided. Moreover, the performance of E2 is better than that of the 2–T TMOS detector. The use of all the considered sensors as proximity detectors has been experimentally verified: the considered detectors therefore represent good solutions for implementing contactless operation of appliances.

## Figures and Tables

**Table 1:** Simulated TMOS sensitivity supposing a 1.2 V supply voltage, an ambient temperature equal to 25 °C and a 10 cm × 10 cm black body at 10 cm distance.

Biasing Current [µA]	Power Consumption [µW]	2–T [µV/°C]	3–T [µV/°C]
0.125	0.15	–32.2	–565.4
0.25	0.3	–31.0	–531.6
0.5	0.6	–29.6	–494.8
1	1.2	–28.3	–456
2	2.4	–27.0	–416.8
4	4.8	–25.8	–384.5
8	9.6	–24.6	–335.8

**Table 2:** Simulated TMOS responsivity.

Biasing Current [µA]	Power Consumption [µW]	2–T [V/W]	3–T [V/W]
0.125	0.15	1.29 × 10${}^{4}$	2.27 × 10${}^{5}$
0.25	0.3	1.24 × 10${}^{4}$	2.13 × 10${}^{5}$
0.5	0.6	1.19 × 10${}^{4}$	1.98 × 10${}^{5}$
1	1.2	1.14 × 10${}^{4}$	1.83 × 10${}^{5}$
2	2.4	1.08 × 10${}^{4}$	1.67 × 10${}^{5}$
4	4.8	1.03 × 10${}^{4}$	1.51 × 10${}^{5}$
8	9.6	0.99 × 10${}^{4}$	1.35 × 10${}^{5}$

**Table 3:** Proposed thermopiles’ measured sensitivity for a 10 cm × 10 cm black body source at 10 cm distance.

Ambient Temperature [°C]	E1 [µV/°C]	E2 [µV/°C]
15	16.6	70.9
25	16.8	68.9
30	17.1	70.1
40	17.5	72.1

**Table 4:** Proposed Thermopiles Measured Responsivity.

E1 [V/W]	E2 [V/W]
4.08 × 10${}^{2}$	2.70 × 10${}^{4}$

**Table 5:** Proposed thermopile sensors’ characteristics.

	E1	E2
Responsivity [V/W]	408	2.70 × 10${}^{4}$
Output Resistance [kΩ]	59.2	947.2
Noise Spectral Density @ 300 K [V/$\sqrt{\mathrm{Hz}}$]	3.13 × 10${}^{-8}$	1.25 × 10${}^{-7}$
Active Area [mm${}^{2}$]	0.4608	0.4608
Detectivity [cm $\sqrt{\mathrm{Hz}}$ W${}^{-1}$]	8.85 × 10${}^{6}$	1.17 × 10${}^{8}$
Response Time [ms]	80	80
Medium	vacuum	vacuum
Device Operating Temperature Range [°C]	–20–85	–20–85
Target Temperature Range [°C]	–20–200	–20–200

## Data Availability

Not applicable.
